# Metnase and EEPD1: DNA Repair Functions and Potential Targets in Cancer Therapy

**DOI:** 10.3389/fonc.2022.808757

**Published:** 2022-01-28

**Authors:** Jac A. Nickoloff, Neelam Sharma, Lynn Taylor, Sage J. Allen, Suk-Hee Lee, Robert Hromas

**Affiliations:** ^1^Department of Environmental and Radiological Health Sciences, Colorado State University, Fort Collins, CO, United States; ^2^Department of Biochemistry & Molecular Biology, Indiana University School of Medicine, Indianapolis, IN, United States; ^3^Division of Hematology and Medical Oncology, Department of Medicine and the Mays Cancer Center, University of Texas Health Science Center, San Antonio, TX, United States

**Keywords:** DNA repair, DNA double-strand breaks, genome instability, homologous recombination, non-homologous end-joining, chromosome decatenation, DNA damage

## Abstract

Cells respond to DNA damage by activating signaling and DNA repair systems, described as the DNA damage response (DDR). Clarifying DDR pathways and their dysregulation in cancer are important for understanding cancer etiology, how cancer cells exploit the DDR to survive endogenous and treatment-related stress, and to identify DDR targets as therapeutic targets. Cancer is often treated with genotoxic chemicals and/or ionizing radiation. These agents are cytotoxic because they induce DNA double-strand breaks (DSBs) directly, or indirectly by inducing replication stress which causes replication fork collapse to DSBs. EEPD1 and Metnase are structure-specific nucleases, and Metnase is also a protein methyl transferase that methylates histone H3 and itself. EEPD1 and Metnase promote repair of frank, two-ended DSBs, and both promote the timely and accurate restart of replication forks that have collapsed to single-ended DSBs. In addition to its roles in HR, Metnase also promotes DSB repair by classical non-homologous recombination, and chromosome decatenation mediated by TopoIIα. Although mutations in Metnase and EEPD1 are not common in cancer, both proteins are frequently overexpressed, which may help tumor cells manage oncogenic stress or confer resistance to therapeutics. Here we focus on Metnase and EEPD1 DNA repair pathways, and discuss opportunities for targeting these pathways to enhance cancer therapy.

## Introduction

DNA damage is a constant threat to genome integrity and numerous DNA damage sensing, signaling, and repair systems help manage these threats, collectively called the DNA damage response (DDR). DNA damage arises spontaneously due to DNA lability, reactive oxygen species generated during oxidative metabolism, activity of various nucleases such as RAG1/2, AID/APOBEC deaminases, and mis-incorporated ribonucleotides (1–7). Exogenous sources of DNA damage comprise physical agents including non-ionizing and ionizing radiation (UV light, X-rays, γ-rays, charged particles), and DNA-reactive chemicals such as alkylating agents and others used as cancer chemotherapeutics (8–11). DNA lesions include ring-opened bases, adducts, inter- and intra-strand crosslinks, protein-DNA crosslinks, and single- and double-strand breaks (DSBs). DSBs are among the most dangerous lesions as they can lead to deleterious mutations (including deletions and insertions), genome rearrangements, and cell death if mis-repaired or unrepaired. The DDR is critical for maintaining genome stability and preventing cancer. The often-altered DDR in cancer cells can thwart the action of anti-tumor chemo- and radiotherapeutics, thus DDR factors are important therapeutic targets (12–14).

DSB sensors and signal transducers include three phosphatidyl inositol 3’ kinase-related kinases (PIKKs), DNA-PKcs, ATM and ATR, and other regulatory factors such as 53BP1, Ku70/Ku80, MRE11-RAD50-NBS1 (MRN), BRCA1, and RIF1 (15). SIRT6, a chromatin-associated protein of the SIRT family of NAD^+^-dependent deacylases and ADP-ribosylases, was recently shown to sense DSBs, promote recruitment of ATM and DSB repair proteins, and promote phosphorylation of histone H2AX (γH2AX) in megabase-pair chromatin domains flanking DSBs (16). PIKK signals can arrest the cell cycle and promote repair, or activate apoptosis of heavily damaged cells (17). Apoptotic signaling by p53 or other checkpoint factors is often dysregulated in cancer, and this promotes tumor cell survival despite significant damage due to endogenous and exogenous stress, i.e., oncogenic stress or genotoxic therapeutics, respectively (18, 19).

**Figure 1:**
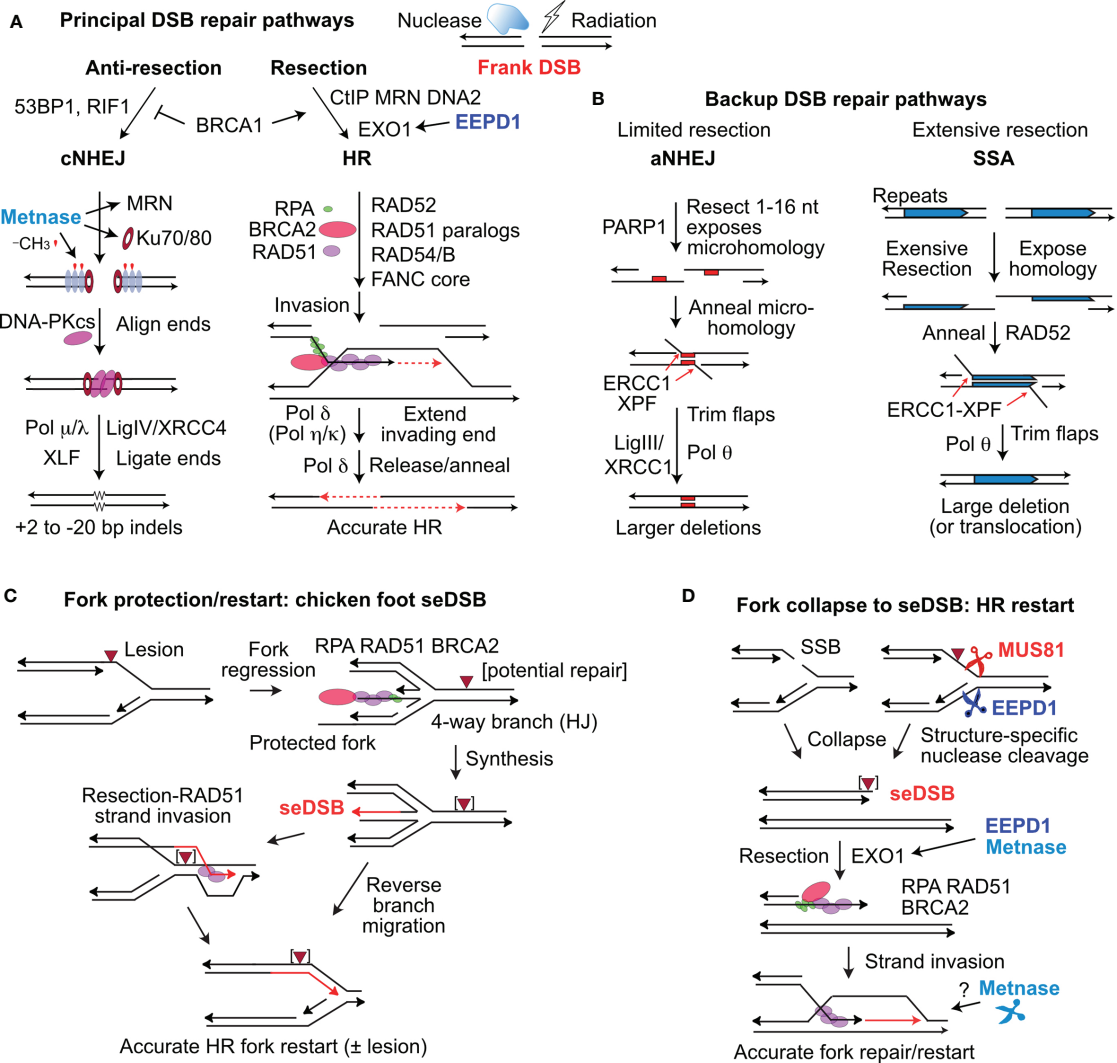
Repair of frank DSBs and seDSBs at collapsed replication forks. **(A)** Nucleases and ionizing radiation create frank, two-ended DSBs processed mainly by cNHEJ and HR regulated by factors that suppress resection (53BP1, RIF1) and those that promote resection (CtIP, MRN, DNA2, and EXO1), controlled by BRCA1. EEPD1 promotes resection through interactions with EXO1. Metnase promotes cNHEJ by methylating histone H3 (red symbols) in nucleosomes (grey ovals) near the DSB, and by promoting recruitment/retention of Ku and MRN. DNA-PKcs interacts with Ku and DNA ends to align ends and promote ligation by DNA ligase IV and other factors. Resected ends are repaired by HR by RAD51 loaded onto resected DNA, mediated by many factors including RPA and BRCA2. RAD51-ssDNA invades homologous duplex DNA and the end is extended (red, dashed arrows), and then released to pair with the second resected end. Gap filling and ligation completes accurate HR repair. **(B)** aNHEJ and SSA are backup repair pathways.  aNHEJ results in larger deletions as ends are aligned at 1-6 nt microhomologies (red rectangles) flanking the DSB exposed by limited resection.  3’ flaps are trimmed by ERCC1-XPF and Ligase III-XRCC1 and Pol θ complete repair that results in loss of one microhomology and intervening sequences.  SSA is analogous to aNHEJ but requires extensive resection to expose repeated sequences that anneal in a RAD52-dependent reaction.  SSA between linked repeats (shown) deletes one repeat and the intervening sequence; SSA between non-linked repeats results in translocations (not shown). **(C)** Forks stalled at blocking lesions can regress to a 4-way branched (chicken foot) structure similar to a Holiday junction (HJ). Extension of the leading nascent strand using the lagging nascent strand as template allows the leading strand to bypass the lesion in the leading template strand. The regressed fork can be restored to a functional fork by reverse branch migration, or by RAD51-mediated strand invasion beyond the blocking lesion. **(D)** Forks may collapse to seDSBs by encountering a single-strand nick, or blocked forks may be cleaved by MUS81 or EEPD1. Resection of the seDSB by EXO1 is promoted by both EEPD1 and Metnase, allowing RAD51-mediated HR to reestablish the fork. Metnase nuclease doesn’t cleave forks, but it may promote HR-mediated fork restart by processing late HR intermediates.

Some DSBs, such as those induced directly by radiation, described as ‘frank’ or ‘two-ended’ DSBs, are repaired by at least four pathways. The two major DSB repair pathways in mammalian cells are classical non-homologous end-joining (cNHEJ) and homologous recombination (HR) (**Figure 1A**). cNHEJ is an error-prone, template-free pathway mediated by Ku70/Ku80, DNA-PKcs, Artemis, DNA polymerases (Pol) μ and λ, XLF, XRCC4, and DNA ligase IV. cNHEJ typically results in small (<20 bp) deletions or short (1-2 bp) insertions (20), but it also mediates translocations if broken ends from different chromosomes are joined (21). HR is generally accurate as it uses a homologous sequence (usually the sister chromatid) as a repair template. ‘Misuse’ of non-sister templates, such as homologous chromosomes or repetitive elements, causes small- and large-scale genome alterations including local loss of heterozygosity by gene conversion, arm-level loss of heterozygosity by inter-homolog crossovers, deletions, inversions, and translocations that are cancer hallmarks (22–24). HR is mediated by RAD51, assisted by BRCA1/2, RAD52, RAD54/B, five RAD51 paralogs (XRCC2, XRCC3, RAD51B, RAD51C, and RAD51D), and the Fanconi anemia proteins (25, 26). End resection is the key determinant of cNHEJ vs. HR pathway choice, regulated by anti-resection factors 53BP1 and RIF1, pro-resection factors BRCA1 and CtIP, and mediated by MRE11, EXO1, and DNA2-BLM (27–32). cNHEJ and HR are backed up by error-prone, alternative NHEJ (aNHEJ) and by single-strand annealing (SSA) (33–36) (**Figure 1B**). aNHEJ requires limited end resection to expose 1-16 nt microhomologies flanking the DSB, although aNHEJ can efficiently join ends with longer ssDNA tails (~50-75 nt) (37, 38). SSA requires more extensive resection to expose long, homologous repeats that are annealed by RAD52 (34); SSA resection tracts >25 kbp have been observed in yeast (39).

A distinct type of DSB arises when replication forks are remodeled or collapse to single-ended DSBs (seDSBs) through fork encounters with single-strand breaks, fork regression to a four-way junction (chicken foot), or when stressed forks are cleaved by structure-specific endonucleases MUS81 or EEPD1 (40–43) (**Figures 1C, D**). An important distinction between frank, two-ended DSBs and seDSBs is that the latter pose significant risk of large deletions or translocations, if repaired by cNHEJ or aNHEJ. Despite these risks, stressed forks are frequently processed to seDSBs by fork regression or fork cleavage (40, 44, 45). Cells have seveal other options to complete DNA replication in the face of replication stress, including rescue of stressed forks by an adjacent fork, translesion synthesis, repriming, and template switching, however, these pathways also pose risks to genome integrity (46–48). Cells prevent genome rearrangements due to misrepair of seDSBs by resecting seDSB ends, which blocks cNHEJ and promotes accurate, HR-mediated fork restart (**Figures 1C, D**) (47). Many of the same HR factors that mediate HR repair of frank DSBs also mediate HR repair of seDSBs to accurately restart collapsed forks. Of note, end resection is critical for HR repair in both repair contexts (26, 47).

Several structure-specific nucleases have been implicated in replication stress responses. The 3’ nuclease MUS81 (with EME1 and EME2 cofactors) cleaves Holiday junction intermediates arising during DSB repair by HR, and stressed replication forks (**Figure 1D**) (40, 41, 49–53). EEPD1 is a 5’ nuclease that cleaves stressed replication forks, complementing the 3’ activity of MUS81 (**Figure 1D**) (42, 43). SLX1, with the SLX4 scaffold protein, cleaves branched DNA structures such as replication forks *in vitro*, but there is no direct evidence that SLX1 cleaves stalled forks *in vivo* (54, 55). Metnase is structure-specific nuclease that promotes restart of stressed replication forks, but Metnase doesn’t cleave stressed replication forks *in vivo*, and may instead process flaps or other branched structures that arise during HR-mediated fork restart (43). Here, we focus on EEPD1 and Metnase roles in DSB repair, replication stress checkpoint activation, restart of stressed forks, and cellular resistance to DNA damaging agents. These topics are discussed with respect to their potential roles in cancer etiology and as therapeutic targets.

## Metnase: A Protein Methyltransferase and Structure-Specific Endonuclease That Promotes DNA Repair in All Cycle Phases

**Figure 2:**
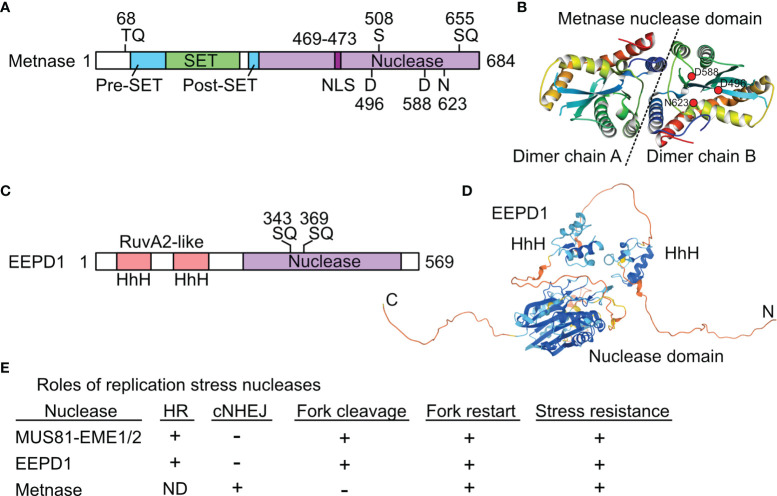
Structures and roles of replication stress nucleases. **(A)** Metnase is a fusion of SET and nuclease domains. Two S/TQ sites (potential PIKK targets) are indicated, along with the DDN nuclease motif. S508 is phosphorylated by Chk1. **(B)** Crystal structure of Metnase nuclease domains shown as a dimer (separated by dashed line), as solved by the Georgiadis lab (59); image from the Protein Data Bank Japan (60) using the Molmil molecular structure viewer (61). Positions of DDN core nuclease residues are indicated in dimer chain B by red dots. **(C)** EEPD1 has two helix-hairpin-helix (HhH) domains related to prokaryotic RuvA2, a component of RuvAB that mediates Holliday junction branch migration. Two potential PIKK target SQ sites are indicated. **(D)** Predicted EEPD1 structure showing HhH and nuclease domains with intervening non-structured regions; image from AlphaFold (62). **(E)** Summary of known functions of three replication stress nucleases. ND, not determined; +, promotes process; -, not involved in indicated process. See text for further details.

Metnase was originally called SETMAR to reflect its SET and *Mariner* lineage (56), but it was renamed Metnase to emphasize its protein methylase and nuclease activities (57). Metnase arose ~50 Mya in monkeys when an Hsmar1 (*Mariner*) transposon integrated downstream of a SET (protein methylase) gene related to human G9a and *Drosophila* Su(var)3-9 and trithorax genes (58), followed by local sequence changes to create the Metnase fusion protein (56) (**Figure 2A**). The crystal structure of the Metnase nuclease domain was solved (59) (**Figure 2B**). Unlike the 200 divergent, non-functional Hsmar1 remnants in the human genome, the Metnase nuclease domain is full length and highly conserved based on an Hsmar1 consensus sequence. This suggests the fusion protein had selective benefits, although the consensus DDD/DDE nuclease active site residues shifted to D_496_D_588_N_623_ in Metnase (58). Both WT (DDN) Metnase and a DDD reconstruction stimulate Hsmar1 transposition *in vitro*, and Metnase binds to Hsmar1 transposon terminal inverted repeat (TIR) sequences (63). Metnase retains only one of two TIRs required for transposition (56), so it cannot mobilize itself.

Initial analysis of Metnase functions demonstrated that it promotes integration of transfected plasmid DNA, cNHEJ, and resistance to ionizing radiation, and that it methylates histone H3 K4 and K36 residues *in vitro*, well-known marks of open chromatin (57). The helix-turn-helix (HtH) motif within the nuclease domain is required for specific binding to TIRs, yet Metnase has non-sequence-specific endonuclease activity that is HtH-independent, but eliminated by a D496A mutation (64) [Note: here we use the aa sequence of the dominant variant, which has 13 more aa than the variant in early publications; thus, D496A is denoted D483A in Roman et al. and other early reports]. Metnase interacts with PSO4 (also known as PRPF19), which functions in transcription-coupled nucleotide excision repair and pre-mRNA splicing (65). PSO4 recruits Metnase to DSBs to promote plasmid DNA integration (65). Metnase also stimulates lentiviral DNA integration (66), consistent with roles in cNHEJ.

Both the Metnase SET and nuclease activities enhance cNHEJ. WT Metnase added to cell extracts promotes cNHEJ, but this activity is eliminated by a nuclease-dead D496A mutation (67), and strongly suppressed by DDD and DDE (Hsmar1-like) versions (68); thus DDN_623_ plays a key role in cNHEJ. Inactivation of the SET domain also abrogates Metnase stimulation of cNHEJ (57). Metnase promotes the efficiency and accuracy of cNHEJ through its interaction with DNA ligase IV (69). Chromosome translocations are often mediated by aNHEJ, and Metnase suppresses translocations by promoting the competing cNHEJ pathway (70). Chromatin immunoprecipitation revealed that Metnase promotes cNHEJ by di-methylating histone H3 K36 in narrow (~2 nucleosome) regions flanking DSBs (71). This contrasts with the far larger γH2AX domains, which extend >1 Mbp from DSBs (~7000 nucleosomes) (72). Importantly, di-methyl H3 K36 near DSBs promotes recruitment and/or retention of early cNHEJ factors Ku70 and NBS1, components of the DNA-PK and MRN complexes, respectively (71). H3 K36 di-methylation is also enhanced at radiation-induced DSBs (71). In cells, DNA repair operates within a chromatin environment, and by the mid-2000s it had been established that chromatin remodeling involving nucleosome eviction by the INO80 complex promotes DSB repair in yeast (73–75); this is also true in mammalian cells (76). The discovery that Metnase promotes cNHEJ by modifying histone H3 adjacent to DSBs was the first suggestion of a histone code for DNA repair (71), analogous to the prototypical histone code for transcription regulation (77).

Metnase also interacts with TopoIIα, which mediates chromosome decatenation of replicated chromosomes before segregation in mitosis. This interaction promotes TopoIIα activity *in vivo* and this activity is suppressed by Metnase automethylation of K495, suggesting that tumors may exploit Metnase to gain resistance to chemotherapeutics that target TopoIIα (78). Indeed, Metnase promotes resistance to the TopoIIα poisons etoposide, doxorubicin, and ICRF-193 in acute myeloid leukemia and breast cancer cells (79, 80). TopoIIα poisons block TopoIIα by binding near its DNA binding site (81). Apparently, Metnase binds this region as well, blocking access and thereby conferring resistance to TopoIIα poisons. Neoamphimedine is a TopoIIα inhibitor derived from a marine sponge that binds near the TopoIIα ATPase domain and thus acts by a different mechanism than traditional TopoIIα poisons. Importantly, Metnase and neoamphimedine bind to distinct regions of TopoIIα, thus Metnase does not confer resistance to neoamphimedine (82).

Metnase SET and nuclease domains play important roles in replication stress responses. siRNA depletion of Metnase delays restart of replication forks stalled by nucleotide depletion with hydroxyurea (HU), and sensitizes cells to HU and several other replication stress agents (83). Fork restart is accelerated by overexpression of Metnase, but this effect is abrogated by defects in the Metnase nuclease or SET domains (68, 84). As noted above, MUS81 and EEPD1 cleave stalled replication forks to promote fork restart *via* HR. Although Metnase accelerates replication fork restart *in vivo*, and cleaves branched structures (including replication forks) *in vitro* (68), Metnase does not cleave stalled forks *in vivo* (43). This raises the possibility that Metnase nuclease promotes fork restart by cleaving flap or other structures that arise during HR-mediated fork restart. Metnase methylation targets during replication fork restart are unknown, but Metnase may methylate histones near stalled forks (43), as it does near DSBs (71). Metnase plays another important role in replication fork restart by HR. Recall that seDSBs at cleaved replication forks must be resected to allow RPA and then RAD51 to bind to 3’ ssDNA tails, which invade sister chromatids to reestablish a functional replication fork (**Figure 1D**). Metnase interacts with EXO1 to promote resection, suppressing cNHEJ of seDSBs and promoting HR-mediated fork restart (85). Metnase is phosphorylated on S508 by Chk1 in response to replication stress; unlike WT Metnase, an S508A mutant does not stimulate cNHEJ, nor does it associate with chromatin in response to replication stress (86). Interestingly, the S508A mutant accelerates replication fork restart more than WT Metnase, suggesting Metnase and Chk1 function in a regulatory feedback loop to coordinate DNA repair and replication stress responses (86). It is intriguing that Metnase promotes cNHEJ of frank DSBs, but suppresses cNHEJ at seDSBs by promoting EXO1 resection at collapsed forks to facilitate HR-mediated fork restart. The lack of cNHEJ activity at seDSBs is reminiscent of the lack of cNHEJ activity by Ku/DNA-PKcs present at telomeres (87). Note that Metnase promotes cNHEJ, a critical function in G1 cells that are largely incapable of HR, it promotes HR-mediated fork restart in S phase, it promotes chromosome decatenation in G2/M phases, and it regulates DNA damage checkpoint signaling. Thus, Metnase augments DNA repair and DDR signaling throughout the cell cycle.

## EEPD1: A Structure-Specific Nuclease That Promotes HR Repair of DSBs and Stressed Replication Forks

EEPD1 was first characterized in 2015. EEPD1 has DNA binding domains with helix-hairpin-helix motifs related to RuvA2, and a DNase I-like nuclease domain (**Figure 2C**). A crystal structure for EEPD1 is not available; a predicted AlphaFold structure (62) is shown in **Figure 2D**. Defects in EEPD1 confer sensitivity to genotoxins, and cause cytogenetic aberrations and cell death by mitotic catastrophe (42, 43). EEPD1 is recruited to and promotes restart of stalled replication forks, and it enhances resection of frank DSBs and seDSBs, thereby suppressing cNHEJ and promoting accurate repair by HR (42). Like Metnase, EEPD1 promotes resection of broken ends through interactions with EXO1, and the resection defects in EEPD1-defective cells prevent ssDNA formation and subsequent activation of ATR and Chk1 (42, 88), indicating that EEPD1 is important for both DSB repair and DNA damage checkpoint signaling. EEPD1 has critical roles during rapid cell proliferation in vertebrate embryonic development (89), highlighting the importance of HR in maintaining genome stability during this sensitive developmental phase. Unlike Metnase, EEPD1 directly cleaves stalled replication forks, similar to MUS81 (42, 43). However, EEPD1 is a 5’ nuclease and MUS81 is a 3’ nuclease. It appears that MUS81, which evolved >1500 Mya in early eukaryotes, was joined by the complementary EEPD1 nuclease in chordates/early vertebrates ~450 Mya. EEPD1 may have been selected to ensure accurate replication of expanding genomes (90) with the consequent increase in replication stress. It is possible that 5’ cleavage of stalled forks by EEPD1 is superior to 3’ cleavage by MUS81 because MUS81 cleaves the leading strand, forcing strand invasion into the lagging (Okazaki) strand which may be delayed until Okazaki fragments mature, and/or further resection occurs to permit HR-mediated fork restart (43). Fork restart timing is important because persistent stalled forks may be restructured into toxic HR intermediates (47, 91), and even short delays in fork restart correlate with increased sensitivity to replication stress and increased genome instability (42, 68, 84, 89). EEPD1 and Metnase both promote HR-mediated fork restart by promoting EXO1 resection of seDSBs, and EEPD1 promotes repair of frank DSBs by HR whereas Metnase promotes frank DSB repair by cNHEJ; there is no evidence that Metnase influences frank DSB repair by HR. The partially overlapping roles of Metnase, MUS81, and EEPD1 in DSB repair and replication stress responses are summarized in **Figure 2E**.

## Metnase and EEPD1 in Cancer Etiology and as Potential Therapeutic Targets

Given their roles in DNA repair, damage signaling, and genome stabilization, it’s possible that defects in Metnase or EEPD1 might predispose to cancer, similar to other DDR factors like BRCA1, BRCA2, and ATM (92). However, no gain or loss of function mutations in Metnase or EEPD1 have yet been verified in cancers; if they exist, they are likely to be rare. Because tumor cells experience considerable stress, i.e., oncogenic stress and DNA damage from therapeutics (93), DDR factors are often overexpressed in cancer. Both Metnase and EEPD1 are frequently overexpressed in breast, brain, cervix, colon, head and neck, kidney, skin, lung, prostate, and uterine cancers; Metnase is also overexpressed in some liver cancers (94). Because these proteins promote tumor cell survival in response to DNA damage by radiation and genotoxic chemotherapeutics, direct inhibition of their nuclease activities, or the Metnase SET activity, may augment traditional chemo- or radiotherapy. Inhibiting Metnase or EEPD1 may be most beneficial for patients whose tumors overexpress these proteins.

There are many cell-based and *in vitro* biochemical assays available to monitor specific Metnase and EEPD1 activities. Defects or inhibition of these proteins uniformly cause sensitivity to genotoxins (42, 57, 83), hence drug screens can be performed using rapid cell survival/proliferation assays (95). If screening for specific nuclease inhibitors, *in vitro* assays with model branched DNA substrates (64, 67), and traditional or automated comet assays (42, 43, 96) are also attractive options. Once a candidate drug is identified, mechanistic insights can be obtained with more time-consuming approaches such as fork restart, chromosome aberration, mitotic catastrophe, and DDR signaling assays.

Current evidence suggests several promising therapeutic approaches. The widely used antibiotic ciprofloxacin inhibits Metnase nuclease and enhances cisplatin sensitivity of A549 lung tumor cells and tumor xenografts in mice (97). TopoIIα poisons are used to treat a variety of tumor types, and tumors that overexpress Metnase may be better controlled with higher doses of traditional TopoIIα poisons (79, 80), or by use of alternative inhibitors (82). Because the Metnase SET activity is important for both cNHEJ and replication fork restart, a specific Metnase SET inhibitor may augment therapeutics that induce frank DSBs and/or replication-associated seDSBs. Although no Metnase SET inhibitors are available, specific SET inhibitors are being developed to treat cancer (98, 99).

Breast and other tumors with BRCA1 or BRCA2 defects are HR-deficient and show synthetic lethality with PARP1 inhibitors, owing to increased replication stress and defective HR-mediated fork restart (100). Inhibition or downregulation of MUS81 also causes synthetic lethality in BRCA2-deficient cells (101). BRCA1, BRCA2, and PALB2 defects are synthetically lethal with RAD52 defects (102, 103), and we found that this lethality depends on EEPD1 (104). Thus, targeting RAD52 may enhance treatment of BRCA-deficient tumors, but co-inhibition of RAD52 and EEPD1 would likely be self-defeating, enhancing tumor cell survival and potentially enhancing tumor progression by allowing severely damaged cells to survive.

Finally, because most cancer therapeutics cause replication stress, combining these agents with inhibitors that target one or more replication stress proteins may improve treatment efficacy. DDR factors including ATR and ATM are being targeted to augment radio- and chemotherapy (105–107). Novel combination therapies targeting upstream PIKKs and/or downstream replication stress nucleases MUS81, EEPD1 or Metnase, may be effective anti-cancer treatments on their own, or when combined with genotoxic chemo- and radiotherapeutics.

## Author Contributions

JN, NS, LT, SA, S-HL, and RH wrote the manuscript and JN prepared the figures. All authors contributed to the article and approved the submitted version.

## Funding

The Nickoloff lab was supported by NIH R01 GM084020 and American Lung Association grant LCD-686552. The Lee lab was supported by NIH R01 CA152367. The Hromas lab was supported by NIH R01 CA205224.

## Conflict of Interest

The authors declare that the research was conducted in the absence of any commercial or financial relationships that could be construed as a potential conflict of interest.

The reviewer EM declared a shared affiliation, with one of the authors, S-HL, to the handling editor at the time of the review.

## Publisher’s Note

All claims expressed in this article are solely those of the authors and do not necessarily represent those of their affiliated organizations, or those of the publisher, the editors and the reviewers. Any product that may be evaluated in this article, or claim that may be made by its manufacturer, is not guaranteed or endorsed by the publisher.
